# Personalized Medicine Based on Nasal Epithelial Cells: Comparative Studies with Rectal Biopsies and Intestinal Organoids

**DOI:** 10.3390/jpm11050421

**Published:** 2021-05-16

**Authors:** Iris A. L. Silva, Violeta Railean, Aires Duarte, Margarida D. Amaral

**Affiliations:** BioISI—Biosystems and Integrative Sciences Institute, Faculty of Sciences, University of Lisboa, 1749-016 Lisboa, Portugal; iasilva@fc.ul.pt (I.A.L.S.); vrailean@fc.ul.pt (V.R.); amduarte@fc.ul.pt (A.D.)

**Keywords:** CFTR, human nasal epithelial cells, rectal biopsies, intestinal organoids, CFTR modulators, theranostic

## Abstract

As highly effective CFTR modulator therapies (HEMT) emerge, there is an unmet need to find effective drugs for people with CF (PwCF) with ultra-rare mutations who are too few for classical clinical trials and for whom there are no drug discovery programs. Therefore, biomarkers reliably predicting the benefit from CFTR modulator therapies are essential to find effective drugs for PwCF through personalized approaches termed theranostics. Here, we assess CFTR basal function and the individual responses to CFTR modulators in primary human nasal epithelial (pHNE) cells from PwCF carrying rare mutations and compare these measurements with those in native rectal biopsies and intestinal organoids, respectively, in the same individual. The basal function in pHNEs shows good correlation with CFTR basal function in rectal biopsies. In parallel, CFTR rescue in pHNEs by CFTR modulators correlates to that in intestinal organoids. Altogether, results show that pHNEs are a bona fide theranostic model to assess CFTR rescue by CFTR modulator drugs, in particular for PwCF and rare mutations.

## 1. Introduction

Cystic fibrosis (CF) is the most common lethal genetic disorder, caused by mutations in the gene encoding for Cystic Fibrosis Transmembrane Conductance Regulator (CFTR), a channel responsible for chloride (Cl^−^) and bicarbonate (HCO_3_^−^) transport in epithelial cells [1,2]. Dysfunctional CFTR leads to the disruption of salts and fluid homeostasis in the cells of the pancreas, airways and intestine, among others [3]. Although CF is a multi-organ disease, progressive loss of lung function is the major cause of morbidity and lethality [4]. In the airways, functional failure of CFTR results in the accumulation of thick and sticky mucus that impedes mucociliary clearance, contributing to chronic bacterial infections and persistent inflammation [5,6].

More than 2100 mutations have been reported so far in the *CFTR* gene [7]. F508del is the most frequent mutation occurring in ~80% of all individuals with CF worldwide and is characterized by defective CFTR processing and trafficking [1]. Currently available CFTR modulators that target the underlying CFTR defect are approved for the treatment of individuals carrying specific CFTR mutations, including F508del [8,9]. However, ~18% of individuals with CF worldwide are not eligible for these therapies since they lack F508del or any other mutation targeted by currently approved CFTR modulator drugs [10]. Moreover, the traditional randomized clinical trials requiring large numbers of individuals to demonstrate drug efficacy become impracticable for those with rare mutations [11,12]. In addition, besides the CFTR genotype, several factors, including modifier genes, environmental factors and lifestyle, are known to modulate the CF disease prognosis and drug response, which highlights the importance of personalized therapy [13,14,15].

Recently, different cellular models have been developed to determine the response of CFTR mutants to modulators through a personalized CFTR pharmacotherapy approach [11]. The most common cellular models include primary human nasal (pHNE) and bronchial (pHBE) epithelial cells and intestinal organoids obtained from rectal biopsies [16,17,18,19,20,21,22,23,24,25,26,27]. Although pHBEs are the gold standard to test for CFTR modulator efficacy since they can recapitulate the in vivo morphology and key processes happening in lungs [28], intestinal organoids are the most advanced model in CF research, incorporating many physiological relevant tissue features [29]. Moreover, the measurement of CFTR-mediated Cl^−^ secretion in human rectal biopsies has been established as a sensitive and robust biomarker for CF diagnosis and prognosis [30,31,32,33,34].

In this work, we aimed to compare CFTR basal function in pHNEs and rectal biopsies from individuals carrying rare mutations, namely P205S, D614G, N1303K, G85E and F508del, and assess whether pHNEs reflect the measurements performed in rectal biopsies in the same individual. Moreover, we also aimed to compare modulator responses in pHNEs and in intestinal organoids also from the same individuals.

Our results show that data in pHNEs significantly correlate with those in both cellular systems, demonstrating that pHNEs are good models both to assess CFTR basal activity and to predict the therapeutic efficacy of CFTR modulator drugs.

## 2. Materials and Methods

### 2.1. Cohort and Ethical Approval

All specimens were obtained from one healthy control and six individuals with CF (carrying F508del/F508del, P205S/Y1092X (two twin sisters), F508del/D614G, F508del/N1303K and G85E/1717-2G>A CFTR genotypes, Table 1) after receiving patient written consent. The study was conducted in accordance with the Declaration of Helsinki, and the protocol was approved by the Ethics Committee of the Santa Maria Hospital and D. Estefânia Hospital (Lisboa, Portugal).

### 2.2. Rectal Biopsies

Human rectal biopsies were obtained by rectoscopy and forceps biopsy as described previously [30,35,36]. The tissue was mounted on a circulating micro-Ussing chamber as described previously [32,33]. The transepithelial measurements were performed under open-circuit conditions in a continuously perfused micro-Ussing chamber at 37 °C, as described previously [30,32,33]. Briefly, the rectal epithelium was continuously perfused (5 mL/min) with Ringer solution with the following composition (mmol/L): NaCl 145, KH2PO4 0.4, K2HPO4 1.6, d-glucose 5, MgCl_2_ 1, Ca-gluconate 1.3, pH 7.40. Tissues were equilibrated in the micro-Ussing chambers for 30 min in perfused Ringer solution before measurements. Amiloride (Amil, 20 μM, luminal) was added to block electrogenic sodium (Na+) absorption through the epithelial Na+ channel (ENaC). Indomethacin (Indo, 10 μM, basolateral) was applied for 20–40 min to inhibit endogenous cAMP formation through prostaglandins. cAMP-dependent and cholinergic Cl^−^ secretion in human rectal tissues relies on functional CFTR. Thus, we used 3-isobutyl-1-methylxantine (IBMX/I, 100 μM, basolateral) and forskolin (Fsk, 2 μM, basolateral) to activate cAMP-dependent Cl^−^ secretion and carbachol (CCH, 100 μM, basolateral) for cholinergic co-activation. In this protocol, Amil was constantly perfused in the luminal side during the experiment.

The parameter that was recently published as being better correlated with disease diagnosis and prognosis was the voltage (Vte) of the maximum activation of CFTR that corresponds to the sum of the IBMX/Fsk and IBMX/Fsk/CCH responses. The percentage of CFTR function was calculated for the average maximal CFTR activation (V_I/F + I/F/C_), normalized to the corresponding mean value determined previously for a reference non-CF control group [30].

### 2.3. Primary Airway Cell Cultures

Primary human nasal epithelial cells (pHNEs) were obtained from nasal brushing as before [37]. After expansion, cells were seeded on porous membranes and cultured under air–liquid interface (ALI) conditions for 21 days following protocols by Jeffrey Beekman’s lab (Utrecht, The Netherlands, submitted to Cell Rep Med). Differentiated monolayers of pHNEs were incubated with 3 µM VX809, 5 µM VX661 (Selleckchem, TX, USA), or DMSO alone for 48 h prior to measurements in an Ussing Chamber. The pHNE monolayers were mounted on micro-Ussing chambers and analyzed under open-circuit conditions, as described previously [16]. CFTR was stimulated by the presence of forskolin (2 µM) + IBMX (100 µM), and specificity was evaluated after using CFTR inhibitor 172 (25 µM). Experiments were performed in the presence of amiloride (20 µM) to inhibit the Epithelial Sodium (Na+) Channel (ENaC) and thus avoid interference of ENaC-mediated currents. In pHNEs, the maximum CFTR activation corresponds to the sum of IBMX/Fsk and IBMX/Fsk+VX-770 responses [16]. CFTR rescue by CFTR modulators in pHNEs was assessed by comparison of the maximum activation of CFTR between control pHNE (DMSO) and treated pHNE (VX809 or VX661). The individuals were considered responders when this difference was statistically different (*p*-value < 0.05). Each experiment was performed at least three times.

### 2.4. Ussing Chamber Measurements

Transepithelial voltage (Vte) values were continuously recorded using Power Lab software (AD Instruments Inc., Dunedin, New Zealand) and referenced to the basal surface of the epithelium. Transepithelial resistance (Rte) was determined by applying short (1 s) intermittent current pulses (0.5 µA). The equivalent short-circuit currents (ΔIeq-sc) were calculated according to Ohm’s law from Vte and Rte (ΔIeq-sc = Vte/Rte), with appropriate correction for fluid resistance [31].

### 2.5. Human Intestinal Organoids Culture

Intestinal organoids were obtained by crypt isolation from rectal biopsies and seeded in 50% Matrigel as described by Vonk et al. [36]. The organoids were passaged ~1 per week, and the medium was refreshed every 2–3 days.

### 2.6. Forskolin-Induced Swelling (FIS) Assay

Forskolin-induced swelling assay was adapted from Dekkers et al. [18]. Briefly, rectal organoids were seeded in a flat-bottom 96-well culture plate in 4 µL of Matrigel and immersed in 50 µL of culture medium, with or without 3 µM VX809 (lumacaftor) or 5 µM VX661 (tezacaftor). After 24 h, the organoids were incubated with 3 µM calcein green (Invitrogen, Waltham, MA, USA) for 20 min; before imaging, they were stimulated with different concentrations of forskolin (0.02, 0.128, 0.8, and 5 µM) (Sigma-Aldrich, St. Louis, MO, USA) alone or in combination with 3 µM VX770 (ivacaftor, Selleckchem, Houston, TX, USA). The organoids were directly analyzed by confocal live cell microscopy (Leica SP8, Leica Microsystems, Wetzlar, Germany) at 37 °C with 5% CO_2_ for 60 min and images were acquired every 10 min. Forskolin-induced organoid swelling was quantified using Cell Profiler software (Broad Institute’s Imaging Platform, Cambridge, MA, USA), image processing and lab-designed scripts [38]. GraphPad Prism was used to obtain and plot values for organoid surface area. Organoid swelling analysis is expressed as the absolute area under the curve (AUC) calculated from the normalized surface area increase (baseline = 100%, *t* = 60 min). Quantification of CFTR response to modulators in organoids was calculated as the difference between non-treated organoids (Fsk) and treated organoids (VX809 or VX661). The individuals were considered “responders” when the AUC was above 1000 after CFTR modulators incubation, as described in Dekkers et al. [18] and Ramalho et al. [39]. Each experiment was performed at least three times and each condition had technical duplicates within the same experiment.

### 2.7. Statistical Analyses

Statistical analyses were performed using one-way ANOVA on GraphPad Prism8 software, and *p*-values of <0.05 were considered significant.

## 3. Results

### 3.1. CFTR Basal Function in Primary Human Nasal Epithelial (pHNE) Cells Correlates with the One Measured in Rectal Biopsies

**Figure 1:**
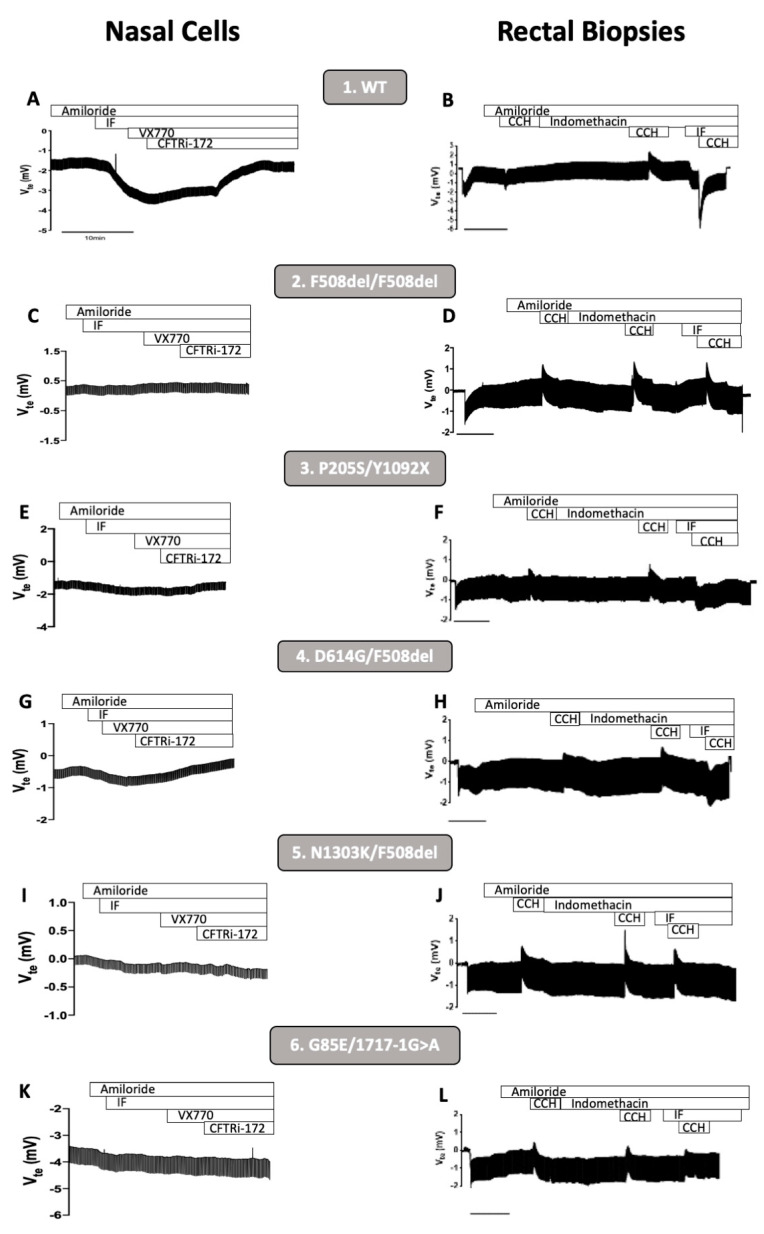
Comparison of basal CFTR-mediated Cl^−^ secretion in primary human nasal epithelial cells (pHNEs) and rectal biopsies. Measurements of basal CFTR-mediated Cl^−^ secretion in pHNEs (left panels) in samples from one healthy control and five PwCF with different genotypes show similarities with the basal CFTR-mediated Cl^−^ secretion measured in rectal biopsies (right panels) from the same individual. The sample code and genotypes are indicated above the tracings. (**A**,**C**,**E**,**G**,**I**,**K**) Original Ussing chamber (open-circuit) recordings showing transepithelial voltage measurements (Vte) obtained for pHNE monolayers with different CFTR genotypes, after cAMP-dependent activation. Amiloride (20 μM) was kept during the whole experiment duration to avoid interference of ENaC-mediated currents. Lumen-negative transepithelial Vte deflections are observed following luminal stimulation by forskolin alone (Fsk, 2 μM) or together with potentiator ivacaftor (VX770, 3 μM). The latter is fully reverted by application of the specific CFTR inhibitor CFTRInh-172 (30 μM). (**B**,**D**,**F**,**H**,**J**,**L**) Representative original recordings of the effects of cholinergic (by carbachol (CCH), 100 μM, basolateral) and cAMP-dependent (by IBMX/Fsk (I/F), 100 μM/2 μM, basolateral) Cl^−^ secretion on transepithelial Vte in rectal biopsies.

In this work, we determined the correlation between CFTR-mediated Cl^−^ secretion measured in pHNEs and in rectal biopsies. Our results show that in non-CF individuals (controls), Fsk induced a lumen-negative deflection which corresponds to the activation of normal CFTR, both in pHNEs and rectal biopsies (Figure 1A,B, individual 1). Additionally, as expected, in samples from a person with CF (PwCF) homozygous for F508del, Fsk failed to induce any CFTR-mediated Cl^−^ secretion in both pHNEs and rectal biopsies (Figure 1C,D, individual 2). By analyzing samples from PwCF with four different rare mutations—P205S, D614G, N1303K, and G85E—we were able to observe that residual CFTR-mediated Cl^−^ secretion was detected both in pHNEs and rectal biopsies from PwCF 3 and 4 with P205S/Y1092X and D614G/F508del genotypes, respectively (Figure 1E–H, individuals 3 and 4). In pHNEs and rectal biopsies from individuals 5 and 6 (Figure 1I–L, individuals 5 and 6) with N1303K/F508del and G85E/1717-1G>A genotypes, respectively, there was a total lack of CFTR-mediated Cl^−^ secretion.

**Figure 2:**
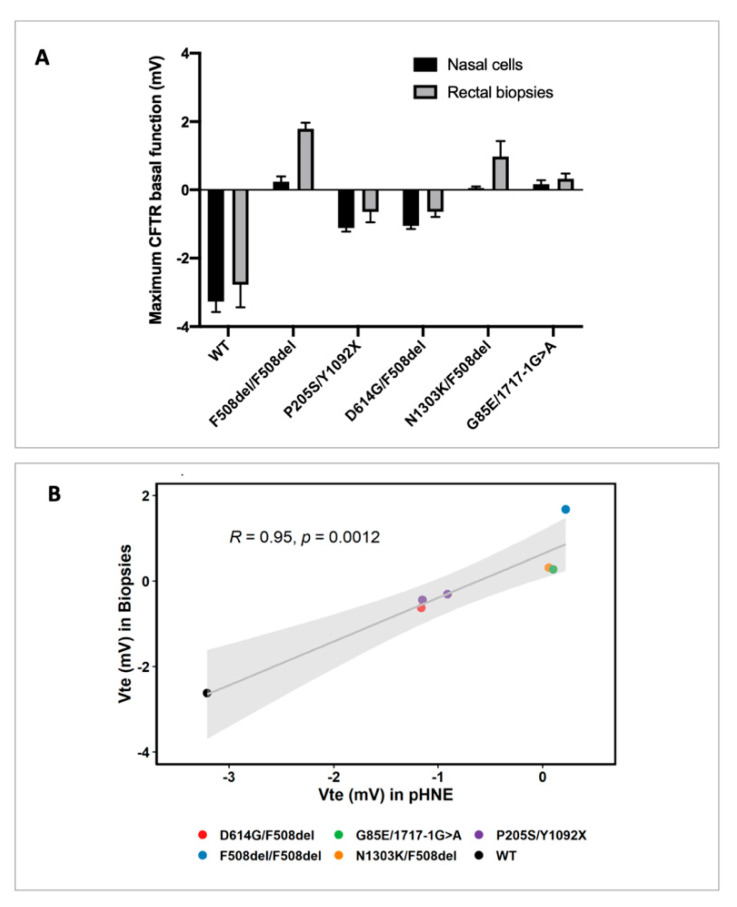
Correlation between maximum activation of CFTR-mediated Cl^−^ secretion in pHNEs and rectal biopsies from the same individual. (**A**) Summary of CFTR-mediated Cl^−^ secretion in rectal biopsies and pHNE cells in one healthy control and five different individuals with CF. CFTR basal function in pHNEs correspond to the differences in Vte after IBMX/Fsk stimulation. CFTR basal function in rectal biopsies is expressed by the difference in Vte upon IBMX/Fsk+IBMX/Fsk/CCH stimulation. Data is represented as the mean of three different experiments for each model ± SD. (**B**) Correlation of CFTR-mediated Cl^−^ secretion stimulated by IBMX/Fsk in pHNEs and IBMX/Fsk+ IBMX/Fsk/CCH in rectal biopsies (Vte_I/F+I/F/C_) from the same individual (Pearson’s R = 0–95, *p*-value = 0.0012). The grey line represents the linear regression of the data. The grey area represents the 95% confidence interval.

We then performed a comparative analysis of CFTR-mediated Cl^−^ secretion in pHNEs and rectal biopsies and observe that whenever residual CFTR-mediated Cl^−^ secretion is present in pHNEs, it is also detected in rectal biopsies (Figure 2A, individuals with WT, P205S/Y1092X, and D614G/F508del genotypes). Similarly, when there is no CFTR-mediated Cl^−^ secretion in pHNEs, it is also not detected in rectal biopsies (Figure 2A, individuals with F508del/F508del, N1303K/F508del, and G85E/1717-1G>A genotypes). In fact, there is a significant correlation of 95% (*p*-value = 0.001) between data obtained in these two models (Figure 2B).

### 3.2. Rescue of CFTR-Mediated Cl^−^ Secretion by CFTR Modulators pHNEs Correlate with CFTR Responses Measured in Intestinal Organoids

Since rectal biopsies cannot be directly used to assess the effect of CFTR modulators, a recent model of intestinal organoids that undergo forskolin-induced swelling (FIS) was developed. In this assay, the swelling of the organoids is directly dependent of CFTR function [18,20]. Thus, we also compared CFTR rescue by CFTR modulators in pHNEs and in intestinal organoids. To this end, intestinal organoids and pHNEs were incubated with CFTR correctors VX-809 or VX-661 for 24 h and 48 h, respectively, for each cellular model.

**Figure 3:**
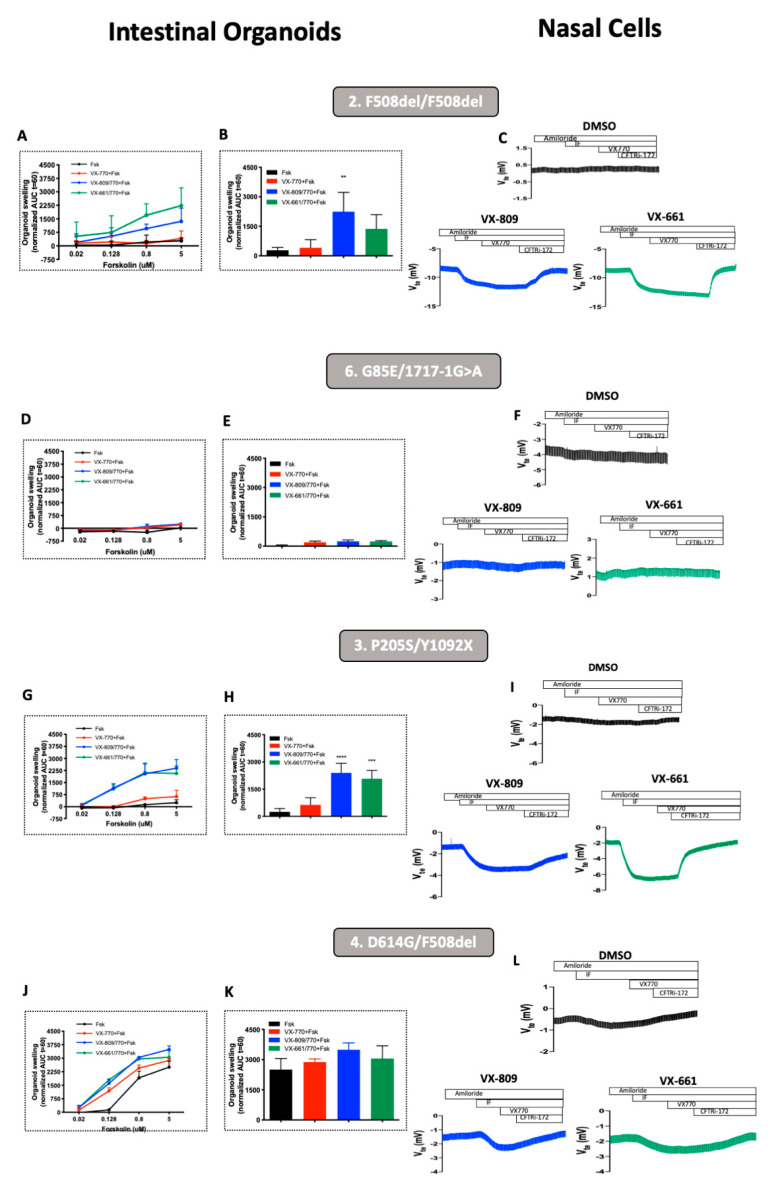
Comparison of CFTR rescue by CFTR modulators in pHNEs and intestinal organoids. Measurements of CFTR rescue by CFTR modulators show similar rescue in pHNEs and intestinal organoids from the same individual. Quantification of forskolin-induced swelling (FIS) assay in intestinal organoids from (**A**) PwCF homozygous for F508del (control, individual 2); PwCF with the (**D**) G85E/1717-1G>A (individual 6); (**G**) P205S/Y1092X (individual 3); and (**J**) D614G/F508del (individual 4) genotypes treated with ivacaftor (VX-770, 3 µM), lumacaftor (VX-809, 3 µM), and tezacaftor (VX-661, 5 µM) at forskolin (Fsk) concentrations of 0.02, 0.128, 0.8, and 5 μM. FIS data are expressed as the area under the curve (AUC) of organoid surface area increase (baseline = 100%, *t* = 60 min). Quantification of these is shown in graphs (**B**,**E**,**H**,**K**) Data represent the mean of measurements ± SD. *p*-value is indicated in the figure (** = < 0.01; *** = < 0.001; **** = < 0.0001); original Ussing chamber (open-circuit) recordings showing CFTR-mediated Cl^−^ secretion measured as transepithelial voltage (Vte) obtained for pHNE monolayers from the same individual (**C**,**F**,**I**,**L**), also treated with DMSO, 3 µM of VX-809 or 5 µM VX-661 for 48 h. Data is represented as the mean of three different technical replicates for each model.

Our results show that, as expected, incubation with VX-809 or VX-661 resulted in CFTR rescue in both the intestinal organoids and pHNEs of PwCF homozygous for F508del (Figure 3A–C). In contrast, no CFTR rescue was observed in the intestinal organoids or pHNEs of the individual bearing the G85E/1717-1G>A genotype (Figure 3D–F). A clear CFTR rescue by CFTR modulators was observed in both the intestinal organoids and pHNEs of the PwCF bearing the P205S mutation (Figure 3G–I, *p*-value < 0.01) when non-treated and treated cells are compared. Although CFTR rescue is clear when analyzing the intestinal organoids of the individual with the D614G mutation at lower concentrations of Fsk, such as 0.128 µM (Figure 3J–L), this effect was not significant when comparing non-treated and treated pHNEs from the same individual (Figure 3H).

**Figure 4:**
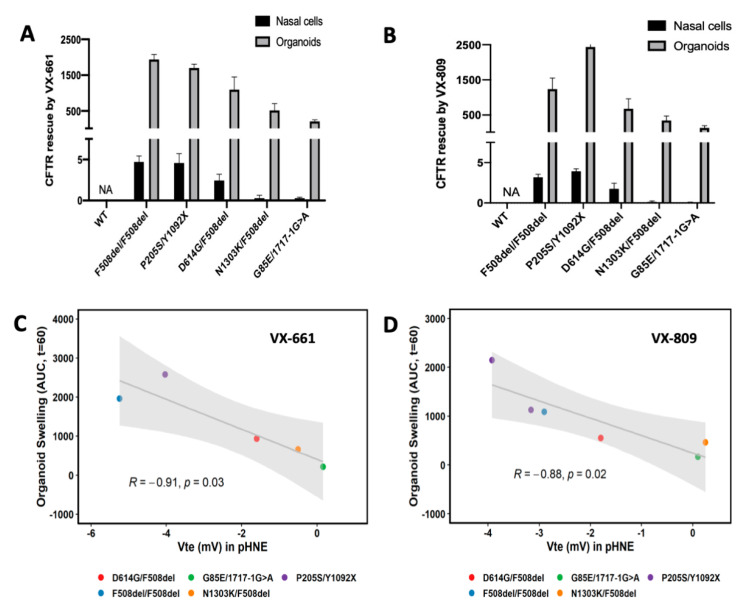
Correlation between CFTR rescue by CFTR modulators in pHNEs and intestinal organoids. (**A**,**B**) Summary of responses in intestinal organoids (shown as ΔAUC (DMSO-treated) *t* = 60, 3 µM VX-809 or 5 µM VX-661) and pHNE cells (shown as transepithelial voltage (Vte)) in five different individuals. WT function in intestinal organoids is not comparable due to pre-swelling (due to CFTR pre-activation by endogenous cAMP). According to standard protocols, pHNEs were treated with DMSO, 3 µM VX-809, or 5 µM VX-661 for 48 h and intestinal organoids for 24 h. Data is represented as the mean of three technical replicates for each model ± SD. (**C**,**D**) Correlation analysis of CFTR rescue in pHNEs and intestinal organoids from the same individual (Pearson’s R = −0.91, *p*-value = 0.03 for VX-661 and Pearson’s R = −0.88, *p*-value = 0.02 for VX-809). The grey line represents the linear regression of the data. The grey area represents the 95% confidence interval.

Next, we performed a comparative analysis of CFTR rescue by CFTR modulators in pHNEs and intestinal organoids. We observe that when we detect CFTR rescue in pHNEs, we also detect it in intestinal organoids, and when there is no rescue of CFTR function in pHNEs, we were also unable to detect it in intestinal organoids (Figure 3, black bars vs. color bars, respectively). Indeed, regarding CFTR rescue by VX-661, when the organoids of PwCF positively respond in a FIS assay (AUC > 1000), the pHNEs of PwCF also display CFTR rescue (Figure 4A). The same is observed for VX-809 (Figure 4B). Finally, the results show that there is a significant correlation of 88–91% (*p*-value = 0.03) between these two models (Figure 4C,D).

## 4. Discussion

As highly effective CFTR modulator therapies (HEMT) emerge, there is an unmet need to find effective drugs for individuals with CF with ultra-rare mutations, for whom there are no dedicated drug discovery programs [3,40,41,42,43,44]. Therefore, the development of techniques and models that reliably predict the clinical benefit from CFTR modulator therapies are required for these individuals through personalized approaches termed theranostics [41]. Here, we assess CFTR basal function and the individual responses to CFTR modulators in primary human nasal epithelial (pHNE) cells from PwCF carrying rare mutations and compare these measurements with those in native rectal biopsies and intestinal organoids.

Our results show a significant and high correlation (95%) between CFTR basal function measured in pHNEs and rectal biopsies (Figure 2). This was somewhat expected since measurements of CFTR-mediated Cl^−^ secretion in both pHNEs and rectal biopsies have already been published by several independent groups and show good correlations with clinical data [16,20,31,33,40]. Additionally, CFTR activation in these two models has some practical similarities since CFTR is activated by the same concentration of Fsk and IBMX (2 µM and 100 µM, respectively) and CFTR activity is measured by the same electrophysiological technique, i.e., in the Ussing chamber. It is important to note that the positive values measured in rectal biopsies, shown in Figure 2A, reflect the cholinergic effect of CCH, which gives rise to luminal positive responses as described by others [30,32,33,45] due to the activation of potassium channels and the lack of CFTR function to compensate for this ion transport.

Here, we also found a significant and high correlation (91%) between CFTR rescue by CFTR modulators in pHNEs and intestinal organoids (Figure 4). This finding highlights that both cellular models can be used to predict the clinical benefit of a single drug or combination of drugs for a certain individual, despite the fact that CFTR function in pHNEs was measured by an Ussing chamber, whereas in intestinal organoids, it was indirectly measured by organoid swelling. Several groups already use either of these two models to predict responses to CFTR modulator drugs [16,17,18,20,39,40,46,47,48,49], but here, we show that the establishment of a responder or non-responder was the same in both models (Figure 3), using both types of samples from the same individual. The only exception was the individual with the D614G/F508del genotype who was considered a non-responder through pHNE analysis but appeared as a responder from intestinal organoid data. This was probably because D614G is a mutation associated with residual CFTR activity and usage of 2 µM of Fsk in pHNEs already over-stimulated CFTR (Figure 3H). This is confirmed by the intestinal organoids results that show at 0.128 µM of Fsk (Figure 3G) a clear difference between the control (black line at 0.128 µM) and the treatments (red, blue, and green lines); however, at 0.8 µM Fsk, that is no longer a significant difference due to high residual function (Figure 3G, black line at 0.8 M). This also stresses the fact that a series of Fsk dilutions is able to better distinguish responders from non-responders.

Regarding the sample collection, we can say that pHNEs are certainly easier to obtain (through nasal brushings) than rectal biopsies and we show that they can reflect the same CFTR activity as rectal biopsies or intestinal organoids. Moreover, other group showed that pHNE are also a surrogate for pHBE [50]. However, there is still a lack of standardized protocols to culture and differentiate these cells and there is a high variability among experimental replicates, even for cells from the same individual cultivated in the same lab. Nevertheless, taken together, these results show that pHNEs from different PwCF with different genotypes can be used to analyze CFTR function and rescue by modulators, in particular for those with rare mutations who cannot undergo clinical trials.

## 5. Conclusions

To conclude, our results show that pHNEs are a bona fide theranostic model to assess CFTR rescue by CFTR modulator drugs, in particular for PwCF bearing rare mutations. Moreover, the assessment of CFTR rescue by modulators should be performed using an individual and personalized approach, where each individual’s cells are incubated with the different modulators available and CFTR rescue should be measured using different techniques that could complement each other (e.g., electrophysiological measurements in an Ussing chamber and FIS assay), aiming at predicting the best treatment that will translate into real clinical benefit.

## Figures and Tables

**Table 1:** Individuals that participated in the study and their respective genotypes.

Individual Code	Genotype
1	WT
2	F508del/F508del
3	P205S/Y1092X
4	D614G/F508del
5	N1303K/F508del
6	G85E/1717-1G>A
7	P205S/Y1092X

## Data Availability

Data available on request due to space restrictions. The raw data presented in this study are available on request from the corresponding author. The data are not publicly available due to space restrictions.

## Abbreviations

AUC	area under the curve
CCH	Carbachol
CF	Cystic Fibrosis
CFTR	Cystic Fibrosis Transmembrane Conductance Regulator
Cl^−^	chloride
ENaC	Epithelial Sodium Channel
Fsk	forskolin
FIS	forskolin induced swelling assay
HCO_3_^−^	bicarbonate
IBMX	3-Isobutyl-1-methylxanthine
pHNE	primary human nasal epithelial cells
pHBE	primary human bronchial epithelial cells
PwCF	people with CF
Vte	Voltage
